# An alternative to the hand searching gold standard: validating methodological search filters using relative recall

**DOI:** 10.1186/1471-2288-6-33

**Published:** 2006-07-18

**Authors:** Margaret Sampson, Li Zhang, Andra Morrison, Nicholas J Barrowman, Tammy J Clifford, Robert W Platt, Terry P Klassen, David Moher

**Affiliations:** 1 Chalmers Research Group, Children's Hospital of Eastern Ontario Research Institute, Canada; 2 Natural Sciences Library, University of Saskatchewan, Saskatoon, Canada; 3 Canadian Agency for Drugs and Technologies, Ottawa, Canada; 4 School of Mathematics and Statistics, Carleton University, Ottawa, ON, Canada; 5 Department of Pediatrics, University of Ottawa, Ottawa, ON, Canada; 6 Department of Epidemiology and Community Medicine, University of Ottawa, Ottawa, ON, Canada; 7 Departments of Pediatrics and of Epidemiology and Biostatistics, McGill University, Montreal, PQ, Canada; 8 Alberta Research Center for Child Health Evidence, Department of Pediatrics, University of Alberta

## Abstract

**Background:**

Search filters or hedges play an important role in evidence-based medicine but their development depends on the availability of a "gold standard" – a reference standard against which to establish the performance of the filter. We demonstrate the feasibility of using relative recall of included studies from multiple systematic reviews to validate methodological search filters as an alternative to validation against a gold standard formed through hand searching.

**Methods:**

We identified 105 Cochrane reviews that used the Highly Sensitive Search Strategy (HSSS), included randomized or quasi-randomized controlled trials, and reported their included studies. We measured the ability of two published and one novel variant of the HSSS to retrieve the MEDLINE-index studies included in these reviews.

**Results:**

The systematic reviews were comprehensive in their searches. 72% of included primary studies were indexed in MEDLINE. Relative recall of the three strategies ranged from .98 to .91 across all reviews and more comprehensive strategies showed higher recall.

**Conclusion:**

An approach using relative recall instead of a hand searching gold standard proved feasible and produced recall figures that were congruent with previously published figures for the HSSS. This technique would permit validation of a methodological filter using a collection of approximately 100 studies of the chosen design drawn from the included studies of multiple systematic reviews that used comprehensive search strategies.

## Background

Search filters or hedges play an important role in evidence-based medicine. For example, work of the HEDGES team has enabled focused clinical searches on PubMed [1] and the original and recently revised highly sensitive search strategy[2] aid in building the evidence base for systematic reviews. Filter validation depends on the availability of a "gold standard" – a reference standard against which to establish the performance of the filter. Our interest is in the development of high recall search filters for systematic reviews[3,4]. We propose an alternative to the traditional gold standard developed through hand searching and we examine the performance of two versions of the Cochrane Highly Sensitive Search Strategy (HSSS)[5] in common usage, as well as a novel version we call the Narrow Boolean Search Strategy (NBSS), against the standard of articles judged relevant for inclusion in systematic reviews.

Recall and precision are the performance characteristics of search strategies that are most relevant to systematic reviews. Recall has as its numerator the number of relevant records in a database retrieved by a search strategy and as its denominator, the total number of relevant records in the database. It is difficult to measure except in experimental conditions. It is widely believed that valid reviews require as complete as possible an identification of relevant studies, thus recall is the most important search parameter from a scientific perspective[6]. Comprehensive searching, with the objective of high recall, is considered standard practice when conducting systematic reviews[7].

Precision has as its numerator the number of relevant records retrieved by a search strategy, and as its denominator, the total number of records retrieved by the search strategy and is thus easily calculated. As precision declines, the burden on reviewers increases as they have more irrelevant items to evaluate for inclusion. Number-needed-to-read is a parameter introduced recently to easily interpret precision figures in the context of systematic reviews – it is the inverse of precision[8].

The ideal search strategy would show high recall and high precision, but there tends to be a trade off between the two, and the relationship typically takes the form of a sigmoid curve[9] rather than the familiar ROC curve seen when sensitivity (recall) is plotted against 1-specificity. Recall and sensitivity are equivalent. Precision is positive predictive value, and is not the same as specificity.

As recall and precision are inversely related, the high recall approach used by systematic reviewers leads to the retrieval of many irrelevant bibliographic records. In practice, restricted by time and cost, reviewers must strive to identify the maximum number of eligible trials, hoping that the studies included in the review will be a representative sample of all eligible studies[10]. The overall time and cost of doing a systematic review depends, in part, on the size of initial bibliographic retrieval[11], thus fine-tuning this initial step in the review process can yield great efficiencies. Cohen *et al*. demonstrated that even modest improvements in precision could save a week's effort in a large review[12]. Methodological filters, sometimes called hedges, attempt to do this by limiting the retrieval to studies of a certain methodological design.

Recall must be calculated to fully evaluate any search strategy, including methodological filters. The real world difficulty in determining the recall of a search strategy is in knowing the denominator. The standard practice amongst those developing search filters for evidence-based medicine has been to establish the gold standard through hand searching of the literature. Jenkins reviewed 20 reports of search filter construction and performance and found that almost all of these filters were validated against a gold standard formed either by hand searching alone, or hand searching and database searching in combination[13]. There are exceptions, particularly when a gold standard collection has been previously assembled. Harrison[14] used a subject bibliography of articles (on evidence-based medicine) as a gold standard. Shojania[15] used existing collections (DARE & reviews published in ACP Journal Club) and Robinson[16] used the CENTRAL database of The Cochrane Library to validate filters. The onerous task of developing the gold standard may be an impediment to filter validation when no definitive collections exist. For instance, the technique of hand searching has been identified as having the lowest yield per time unit of the all commonly used methods for identifying studies for systematic reviews[17].

An approach to filter validation that has not been used widely in the filter development literature is relative recall. Relative recall is the proportion that any specific system retrieves of the total or pooled relevant documents retrieved by all systems considered to be working as a composite[18]. Systematic reviews commonly use multiple systems of retrieval within each review. Each database searched and each non-database strategy (such as contacting experts, reviewing of reference lists, and hand searching journal issues) can be considered as an approach to the literature. Each of these approaches or retrieval systems will differ in terms of recall and precision.

The relative recall technique that we explore in this paper has been used by a number of authors in the context of information retrieval in evidence-based healthcare, although the term relative recall may not have been used. For example, Vincent *et al*. used the technique, describing it as a pragmatic approach, in developing search filters for diagnostic studies on deep vein thrombosis. They used the included studies of 16 published systematic reviews on the topic as a reference set in order to establish a reference set with a broader range of journals and publication years than could have been practically achieved through hand searching[19]. Similarly, Doust *et al*. used relative recall as the basis for comparison of 5 methodological filters for identifying diagnostic studies. The filters were tested against the included studies for 2 systematic reviews in different fields[20]. They suggest that while relative recall may over-state sensitivity (recall) if some studies were missed by the searches for systematic reviews, it is preferable to a hand searching gold standard based on highly selected journals, as it may be more generalizable to topics where the literature is spread across a broad range of journals.

Other examples of its use are Wieland *et al*. who used the technique in a preliminary study to design a filter for maximally sensitive MEDLINE search for observational studies of a relationship between an exposure and disease. The reference standard was the 58 included studies of a single systematic review[21]. Hersh approximated recall of an automated retrieval system (SAPHIRE) using the recall of 3 or more searchers against a MEDLINE test collection as the gold standard[22]. McKibbon *et al*. used the technique to calculate recall in a study assessing MEDLINE searches by clinical end-users and librarians, and used the term relative recall to describe it. The basis for recall was the number of relevant citations retrieved from an individual search divided by the total number of relevant citations from all searches on the same topic.

We explore relative recall as a general technique for forming a reference standard (gold standard) for use in evaluating search filters. In the course of doing a systematic review, reviewers assess items identified from all sources for relevance, and relevant studies are included in the review. The success of any one system used in the review, compared to the pooled relevant documents (included studies) retrieved by all systems working as a composite, is the relative recall of that system.

There are some limitations to relative recall, and these are well described by Fricke[18]. The most important limitation for our purposes is that relative recall is only as good as the sum of the individual searches. It is theoretically possible to search a number of sources and still fail to retrieve an important number of relevant items, and von Tulder *et al*. present a case study of such a situation[23]. The relative recall of one system may be high, yet the actual recall could be close to zero[18]. To guard against this possibility, we propose determining search performance across a number of systematic reviews, and we demonstrate the approach using several variants of a well-established filter, the Highly Sensitive Search Strategy (HSSS).

Since its introduction in early 1990's, the HSSS has been widely used by information specialists and medical practitioners to find reports of randomized controlled trials (RCTs) in MEDLINE. The complete HSSS contains three phases [see Additional file 1]. We refer to the full search, with all three phases, as HSSS_123 _and the two-phase search as HSSS_12_. The HSSS_12 _was selected for searching all of MEDLINE in the retagging project when a pilot study conducted by the UK Cochrane Centre in 1994 concluded that the terms in the third phase of the HSSS was too broad for use without a subject search[24]. In an assessment of the HSSS_123_, McDonald found precision to be 7.8% for the years 1994–1997, and reported the precision of individual terms[25].

## Methods

### Modification of the HSSS

To create an additional strategy for comparison, we used the precision of individual terms reported by McDonald to form a more concise search strategy, removing some of the terms with low precision from the HSSS. We call this variant the Narrow Boolean Search Strategy (NBSS) [see Additional file 1].

### Formation of a reference set of systematic review

#### Data sources

The Cochrane Database of Systematic Review 3^rd ^Quarter 2002 was searched through the Ovid interface to identify systematic reviews that appeared to use elements of the HSSS [see Additional file 1 for the search strategy].

#### Eligibility criteria

To be eligible, a systematic review had to use at least two sections of the Highly Sensitive Search Strategy to find RCTs in MEDLINE. Specifically, we excluded reviews that did not report using the HSSS, that reported using only the first section, or when they reproduced the MEDLINE search strategy, did not reproduce at least 2 sections of the HSSS. We accepted reviews that stated that they used the HSSS, without specifying which sections. In addition to using the HSSS, the systematic review must have reported the citations for included studies. Finally, the review must have had as an inclusion criterion that primary studies were either RCT or quasi RCT. Two reviewers (MS, AM) examined each systematic review for eligibility. Conflicts between the two reviewers were resolved through consultation and consensus.

#### Data extraction

For each eligible systematic review, a known-item search for each included study was undertaken to determine if it was indexed in MEDLINE or not. Searching was completed by a single librarian (LZ) between January 2003 and April 2003, using the Ovid interface for MEDLINE 1966–2003. For an example of known item search see Additional file 1. The included trials of each systematic review that were found in MEDLINE were aggregated using the Boolean operator OR. The included trials were combined with the HSSS_12_, the HSSS_123_, and NBSS to determine the number of trials that were retrieved by each. The number of included trials cited in a systematic review and the number of included trials indexed in MEDLINE was recorded. Additional characteristics of the systematic reviews and the search strategy were extracted by MS.

#### Calculation of relative recall

We define relative recall as:

Relative Recall=No. of included trials of each SR retrieved by a search strategyNo. of included trials of each SR indexed in Medline
 MathType@MTEF@5@5@+=feaafiart1ev1aaatCvAUfKttLearuWrP9MDH5MBPbIqV92AaeXatLxBI9gBaebbnrfifHhDYfgasaacH8akY=wiFfYdH8Gipec8Eeeu0xXdbba9frFj0=OqFfea0dXdd9vqai=hGuQ8kuc9pgc9s8qqaq=dirpe0xb9q8qiLsFr0=vr0=vr0dc8meaabaqaciaacaGaaeqabaqabeGadaaakeaacqqGsbGucqqGLbqzcqqGSbaBcqqGHbqycqqG0baDcqqGPbqAcqqG2bGDcqqGLbqzcqqGGaaicqqGsbGucqqGLbqzcqqGJbWycqqGHbqycqqGSbaBcqqGSbaBcqGH9aqpdaWcaaqaaiabb6eaojabb+gaVjabb6caUiabbccaGiabb+gaVjabbAgaMjabbccaGiabbMgaPjabb6gaUjabbogaJjabbYgaSjabbwha1jabbsgaKjabbwgaLjabbsgaKjabbccaGiabbsha0jabbkhaYjabbMgaPjabbggaHjabbYgaSjabbohaZjabbccaGiabb+gaVjabbAgaMjabbccaGiabbwgaLjabbggaHjabbogaJjabbIgaOjabbccaGiabbofatjabbkfasjabbccaGiabbkhaYjabbwgaLjabbsha0jabbkhaYjabbMgaPjabbwgaLjabbAha2jabbwgaLjabbsgaKjabbccaGiabbkgaIjabbMha5jabbccaGiabbggaHjabbccaGiabbohaZjabbwgaLjabbggaHjabbkhaYjabbogaJjabbIgaOjabbccaGiabbohaZjabbsha0jabbkhaYjabbggaHjabbsha0jabbwgaLjabbEgaNjabbMha5bqaaiabb6eaojabb+gaVjabb6caUiabbccaGiabb+gaVjabbAgaMjabbccaGiabbMgaPjabb6gaUjabbogaJjabbYgaSjabbwha1jabbsgaKjabbwgaLjabbsgaKjabbccaGiabbsha0jabbkhaYjabbMgaPjabbggaHjabbYgaSjabbohaZjabbccaGiabb+gaVjabbAgaMjabbccaGiabbwgaLjabbggaHjabbogaJjabbIgaOjabbccaGiabbofatjabbkfasjabbccaGiabbMgaPjabb6gaUjabbsgaKjabbwgaLjabbIha4jabbwgaLjabbsgaKjabbccaGiabbMgaPjabb6gaUjabbccaGiabb2eanjabbwgaLjabbsgaKjabbYgaSjabbMgaPjabb6gaUjabbwgaLbaaaaa@D087@

We use the number of included trials in MEDLINE as the denominator because this represents the composite pool of items available to our filters.

## Results

**Figure 1:**
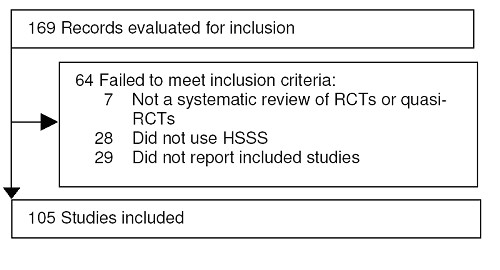
QUOROM Flow Diagram.

169 systematic reviews in Cochrane Database of Systematic Reviews were identified for screening. 64 of these were excluded either because they did not use RCTs or quasi RCTs as the basis for inclusion in the review (n = 7), did not appear to use any part of HSSS (n = 28) or they do not report the citations of included and excluded trials (n = 29). 105 systematic reviews meet all inclusion criteria (Figure 1). See Additional file 2 for a listing of included studies.

Reviews were drawn from the 3^rd ^Quarter 2002 issue of The Cochrane Library and most were of recent origin. The vast majority were reviews of treatment interventions. Over half included RCTs exclusively. Although all specified RCT or quasiRCT as an inclusion criterion, a number included other controlled clinical trials. Thirty Cochrane review groups were represented. The 10 groups having 4 or more included reviews accounted for 71% of reviews in the sample (Table 1). The reviews had a median of 12 included studies, somewhat larger than the review described as typical by Mallette and Clarke[26], which had a median of 6 included studies (interquartile range 3–12).

**Table 1:** Characteristics of the Included Systematic Reviews

Characteristic	n (total = 105)	%
Year of publication or substantive update		
Median	2001	
Interquartile range	1999–2001	
		
Focus of the review		
Treatment	89	84.8
Prevention	14	13.3
Diagnosis	1	1.0
Other	1	1.0
		
Study designs included		
RCT only	55	52.4
RCT and qRCT	39	37.1
RCT and other controlled trials	11	10.5
		
Meta-analysis undertaken	78	74.3
		
Review groups with 4 or more included reviews*		
Musculoskeletal Injuries	22	21.0
Eyes and Vision	12	11.4
Renal	9	8.6
Prostatic Diseases and Urologic Cancers	8	7.6
Back	6	5.7
Schizophrenia	6	5.7
Depression, Anxiety and Neurosis	5	4.8
Infectious Diseases	4	3.8
Upper Gastrointestinal and Pancreatic Diseases	4	3.8
Other	29	27.6
		
Number of included studies per review		
Median	12	
Interquartile range	5.0–19.5	

Other group representation is as follows: Menstrual Disorders, Skin (3 reviews each), Effective Practice and Organisation of Care, Gynaecological Cancer, Heart, Hepato-Bilary, Hypertension, Lung Cancer (2 reviews each), Acute Respiratory Infections, Colorectal Cancer, Drugs and Alcohol, Ear, Nose and Throat, Fertility Regulation, HIV/AIDs, Metabolic and Endocrine Disorders, Movement Disorders, Oral Health, Pregnancy and Childbirth, Stroke (1 review each).

Searches were comprehensive, using multiple electronic sources and traditional techniques to identify relevant reports. The median of included trials indexed in MEDLINE was 9 (interquartile range 4–17) (Table 2).

**Table 2:** Characteristics of the Searches

Characteristic	N (total = 105)	%
Number of electronic databases searched:		
Median	5	
Interquartile range	3–6	
		
Number of non-database techniques used:		
Median	2	
Interquartile range	2–3	
		
Phases of the HSSS used:		
1 and 2	44	41.9
1,2 and 3	30	28.6
Unspecified	31	29.5
		
Number of included studies indexed in MEDLINE:		
Median	9	
Interquartile range	4–17	

Eighty different electronic sources were mentioned in the 105 reviews. Major electronic sources (cited in 10% or more of reviews) and all non-electronic sources are shown in Table 3. In addition, there were 17 other electronic sources that were reported used by 5 or more reviews. 54 different electronic sources were used in less than 5 reviews. Of those, 32 of those were mentioned in only one review. These results are similar to those of Royle and Milne based on a sample of Cochrane reviews published for the first time in the Cochrane Library 2001, Issue 1 [27] and there is most likely some overlap between their sample and ours.

**Table 3:** Sources Searched

**Electronic sources searched in at least 10% of reviews**	**N**	**%**
MEDLINE	105	100.0
Embase	93	88.6
Cochrane Controlled Trials Register or CENTRAL or review group registry	91	86.7
Science Citation Index or Social Science Citation Index	37	35.2
CINAHL	31	29.5
PsycLit	18	17.1
Cochrane Library (no section specified)	16	15.2
Biosis	12	11.4
Dissertation Abstracts	12	11.4
National Research Register	11	10.5

**Non-electronic search techniques (all reported methods)**		

Cited references	91	86.7
Experts and/or authors or non-commercial organizations contacted	67	63.8
Contacted industry (pharmaceutical companies, manufacturers)	30	28.6
Hand searching (other than reference lists)	17	16.2
Own files	2	1.9
Print indices other than Index Medicus	2	1.9
Index Medicus	1	1.0
Subject Bibliography	1	1.0

### Relative recall

Among the 2014 included trials, 1456 were found, through known item searching, to be indexed in MEDLINE at the time of our searching (72%). We use this as the denominator in our calculation of relative recall. Using HSSS_123 _would have resulted in the retrieval of 1422 of these (relative recall = .98), HSSS_12 _retrieved 1370 of these items (relative recall = .94), and NBSS retrieved 1322 of these (relative recall = .91).

Looking at performance of the various filters in individual reviews, of our collection of 105, HSSS_123 _retrieved all MEDLINE included studies in 86 reviews, HSSS_12 _in 72 reviews, and NBSS in 57. One extreme case was found in which all three search strategies missed a number of included items[28] however, in the case with the most included studies indexed in MEDLINE (n = 103), all three search strategies retrieved all 103 items[29]. The distribution of number of misses on a review-by-review basis is presented in Table 4.

**Table 4:** Frequency of misses on a per review basis, three strategies compared

	Number of reviews with items in MEDLINE, but missed		
Number of items missed	HSSS_123_	HSSS_12_	NBSS
1	14	19	23
2	2	2	7
3	2	6	8
4	-	2	4
5	-	1	2
6	-	1	2
			
10	1*	1	-
			
12	-	-	1
			
16	-	1*	-
			
23	-	-	1*

N of reviews with missing items	19	33	48
Median missed items per review	0	0	0
Interquartile range	0 – 1	0 – 1	0 – 0

* These misses are attributable to review #129, Wilhelmus KR. Interventions for herpes simplex virus epithelial keratitis. Cochrane Database of Systematic Reviews 2002; The Cochrane Library, Issue 3, 2002. This review had 146 included studies, of which 89 could be found in MEDLINE. 73 of these were retrieved by the HSSS12, 66 by the NBSS. An additional 6 could have been retrieved by phase 3 of the HSSS, which the authors did not report using.

## Discussion

Our results support the use of relative recall as a filter validation technique by those without the resources for extensive hand searching. Our figures for recall are somewhat higher than the 0.80 recall of HSSS reported by Hopewell[30]. If studies not indexed in MEDLINE are included in the denominator, as appears to be the case in some of the studies considered by Hopewell, our recall figures for the three search variants would decline to 0.701 for the HSSS_123_, 0.68 for the HSSS_12_, and 0.656 for the NBSS.

We also see our results behave as expected across the three variants tested here. The three strategies are variants on a theme. The HSSS_123 _is the broadest. HSSS_12 _is narrower, as fewer terms are joined with Boolean OR and no new terms are introduced. NBSS is narrower again. As expected, the three phases gave the highest recall, there was a 4 point drop when only phase 1 and 2 were used and a further 3 point drop if the narrower search tested here were used. The HSSS_12 _provided high relative recall overall and recall as good as the full three section HSSS_123 _in 72 cases (69%). The NBSS that we introduce here appears to result in a relatively small decline in recall, relative to broader searches. This could be interpreted as providing some support for the technique of picking terms based on performance of individual terms (i.e in the McDonald paper), although the resulting search appears insufficiently sensitive for use in the systematic review context due to the need to maximize recall.

Our findings are based on a large number of reviews from 30 review groups corresponding to different medical specialties. Most of these reviews featured comprehensive searches, using both electronic and non-electronic sources. Thus the composite search result of the individual studies is apt to be quite robust. The pooling of included studies from many reviews helps mitigate against errors or ineffective search performance of individual reviews, and supports the generalisability of these results across subject areas. Thoroughness of the composite search, in terms of number of systems searched and the adequacy of those searches, varied between reviews in our sample. To use this technique of relative recall to validate new filters for other research designs, we suggest restricting the sample to those meeting some minimum criteria for completeness. We also caution that most reviews in this study (87%) benefited from a search of CENTRAL – a comprehensive register of RCTs[31]. Designs other than RCTs do not benefit from the existence of such a comprehensive register – a "system" included in many of these searches, and that might lower the real world recall of the composite searches and so the completeness of the reference set. Further, almost all reviews examined here studied intervention effectiveness. This technique may not generalize to searches for diagnostic reviews.

The main advantage of using a relative recall approach instead of a gold standard developed through hand searching is efficiency. How many studies of the design sought might be needed for the reference standard created by pooling the included studies of systematic reviews? Sample size calculation are rarely reported in studies of filter development[13] but are needed to establish the confidence interval around the estimate of recall. Search performance figures (recall and precision) can be treated as proportions, for the purpose of establishing confidence intervals and it can be shown that the confidence interval decreases, for a given sample size, as the true recall increases. The mean sensitivity (recall) reported in the search filters considered by Jenkins was .936 (standard deviation 7.07) and median = 96.9 (interquartile range .90–.99). If the desired recall of a new search strategy is 0.9, a sample of about 100 included studies of the appropriate design would be needed to establish a 95% confidence interval of .84–.96. This translates into 4–16 missed studies per 100. This is a slight oversimplification because the confidence interval becomes asymmetric as it approaches 1, and so a confidence interval of .82–.94 may be more realistic. By comparison, if the true recall is .5, the margin of error becomes +/- 10%, or 40 to 60 studies missed per 100.

The feasibility of the relative recall approach we have described for validating novel methodological filters may depend on the availability of a number of systematic reviews using the methodology of interest. In the example above, 100 included studies of a given design would be needed, from several systematic reviews.

The relative recall approach as a gold standard has the added advantage of taking into account user preference, that is, the assessment by expert reviewers that the item is indeed relevant for the review. In addition to providing a gold standard for evaluating searches, relative recall can also be used in testing the contribution of databases[13,32].

This paper has focused on recall and precision as the basis for evaluation of search performance. Numerous criticisms of these measures have been made, and these are well reviewed by Kagolovsky and Moehr[33] Never-the-less, the information retrieval paradigm used in systematic reviews is classically suited to evaluation using the measures of recall and precision. Retrieval occurs in batch mode, although preliminary work may be exploratory and interactive. High recall and high precision are sought, with large retrieval sets being the norm. Retrieved documents are classified into a binary relevance scheme as eligible or ineligible for inclusion in the review. Finally, measures are taken to minimize the subjectivity or idiosyncrasy of the relevance assessment: the search result is evaluated against explicit criteria, often by 2 reviewers who much reach consensus, so that the work could be independently replicated.

While we propose relative recall as an alternative to hand-searching in the formation of a gold standard for search strategy development, our methods are indirect. A useful avenue for further study would be such a direct comparison between a standard based on the included studies of systematic reviews and one derived from hand searching (or, with the growth in availability of electronic full text articles, on-screen searching). The most useful comparison would not only examine the information retrieval characteristics of the two approaches, but would also compare the resources required to assemble the collections.

Finally, it must be underscored that known item searching of MEDLINE retrieved only 72% of included studies. While the proportion of included studies indexed in MEDLINE is higher than the 51% sensitivity of MEDLINE reported by Dickersin[5] it is similar to the 68.8% inclusion reported by Royle and Milne[27]. This result, while not novel, reinforces the need for multifaceted searching in order to identifying all relevant primary studies for systematic reviews.

## Conclusion

The relative recall approach of using included studies of a certain design, pooled across a number of reviews appears to be a promising alternative to hand searching for those wishing to develop a reference standard for new methodological filters. It may be possible to validate methodological filters based on approximately 100 included studies of the desired design drawn from a number of systematic reviews with comprehensive searches.

## Competing interests

The author(s) declare that they have no competing interests.

## Authors' contributions

*MS *conceptualized the project, screened records for eligibility, undertook data collection and analysis, prepared the first draft of the manuscript, and participated in all revisions. *LZ *undertook the searching, created the datasets, and participated in all revisions of the manuscript. *AM *screened records for eligibility, undertook data collection, and participated in all revisions of the manuscript. *NJB *obtained funding for the project, designed the statistical analysis, and participated in the drafting and revision of the manuscript. *DM *obtained funding for the project, advised on the design and conduct of the research and participated in all revisions of the manuscript. *TJC *acted as project leader for the grant, advised on the design and conduct of the research and participated in all revisions of the manuscript. *RWP *obtained funding for the project, advised on the statistical analysis, the design and conduct of the research and participated in all revisions of the manuscript. *TK *obtained funding for the project, the design and conduct of the research and participated in all revisions of the manuscript

## Pre-publication history

The pre-publication history for this paper can be accessed here:



## Supplementary Material

Additional File 1 Appendix 1 Search Strategies Used. Appendix listing search strategies. Click here for file

Additional File 2 Appendix 2 – Included Studies (n = 105). List of studies included in this review. Click here for file
