# Supplementary material for: An alternative to the hand searching gold standard: validating methodological search filters using relative recall
Source: BMC Med Res Methodol. 2006 Jul 18;6:33. doi: 10.1186/1471-2288-6-33 (PMC1557524; doi:10.1186/1471-2288-6-33)
Supplement: Additional File 1 — Appendix 1 Search Strategies Used. Appendix listing search strategies. [file 1471-2288-6-33-S1.doc]

### Appendix 1 Search Strategies Used

### Strategy for identifying candidate systematic reviews in Cochrane Database of Systematic Reviews:

1. Hsss.tw.

2. highly sensitive search.tw.

3. 1 or 2

### HSSS_12_

1. RANDOMIZED CONTROLLED TRIAL.pt.
2. CONTROLLED CLINICAL TRIAL.pt.
3. RANDOMIZED CONTROLLED TRIALS.sh.
4. RANDOM ALLOCATION.sh.
5. DOUBLE BLIND METHOD.sh.
6. SINGLE-BLIND METHOD.sh.
7. or/1-6
8. (ANIMAL not HUMAN).sh.
9. 7 not 8
10. CLINICAL TRIAL.pt.
11. exp CLINICAL TRIALS/
12. (clin$ adj25 trial$).ti,ab.
13. ((singl$ or doubl$ or trebl$ or tripl$) adj25 (blind$ or mask$)).ti,ab.
14. PLACEBOS.sh.
15. placebo$.ti,ab.
16. random$.ti,ab.
17. RESEARCH DESIGN.sh.
18. or/10-17
19. 18 not 8
20. 19 not 9
21. 9 or 20

### HSSS_123_

1. RANDOMIZED CONTROLLED TRIAL.pt.

2. CONTROLLED CLINICAL TRIAL.pt.

3. RANDOMIZED CONTROLLED TRIALS.sh.

4. RANDOM ALLOCATION.sh.

5. DOUBLE BLIND METHOD.sh.

6. SINGLE-BLIND METHOD.sh.

7. or/1-6

8. (ANIMAL not HUMAN).sh.

9. 7 not 8

10. CLINICAL TRIAL.pt.

11. exp CLINICAL TRIALS/

12. (clin$ adj25 trial$).ti,ab.

13. ((singl$ or doubl$ or trebl$ or tripl$) adj25 (blind$ or mask$)).ti,ab.

14. PLACEBOS.sh.

15. placebo$.ti,ab.

16. random$.ti,ab.

17. RESEARCH DESIGN.sh.

18. or/10-17

19. 18 not 8

20. 19 not 9

21. COMPARATIVE STUDY.sh.

22. exp EVALUATION STUDIES/

23. FOLLOW UP STUDIES.sh.

24. PROSPECTIVE STUDIES.sh.

25. (control$ or prospectiv$ or volunteer$).ti,ab.

26. or/21-25

27. 26 not 8

28. 27 not (9 or 20)

29. 9 or 20 or 28

### NBSS

1. (Double blind$ or single blind$).mp.
2. Placebo$.ti,ab.
3. placebos.sh.
4. random$.ti,ab.
5. random allocation.sh.
6. randomized controlled trials.sh.
7. Randomized controlled trial.pt.
8. or/1-7
9. (ANIMAL not HUMAN).sh.
10. 8 not 9
