# Supplementary material for: An alternative to the hand searching gold standard: validating methodological search filters using relative recall
Source: BMC Med Res Methodol. 2006 Jul 18;6:33. doi: 10.1186/1471-2288-6-33 (PMC1557524; doi:10.1186/1471-2288-6-33)
Supplement: Additional File 2 — Appendix 2 – Included Studies (n = 105). List of studies included in this review. [file 1471-2288-6-33-S2.doc]

### Appendix 2 - Included Studies (n=105)

Alejandria MM, Lansang MA, Dans LF, Mantaring JBV. Intravenous immunoglobulin for treating sepsis and septic shock. Cochrane Database of Systematic Reviews 2002; The Cochrane Library, Issue 3, 2002.

Andersen BR, Kallehave FL, Anderson HK. Antibiotics versus placebo for prevention of postoperative infection after appendicectomy. Cochrane Database of Systematic Reviews 2002; The Cochrane Library, Issue 3, 2002.

Athaullah N, Proctor M, Johnson NP. Oral versus injectable ovulation induction agents for unexplained subfertility. Cochrane Database of Systematic Reviews 2002; The Cochrane Library, Issue 3, 2002.

Bagnall AM, Lewis RA, Leitner ML. Ziprasidone for schizophrenia and severe mental illness. Cochrane Database of Systematic Reviews 2002; The Cochrane Library, Issue 3, 2002.

Bagnall AM, Fenton M, Lewis R, Leitner ML, Kleijnen J. Molindone for schizophrenia and severe mental illness. Cochrane Database of Systematic Reviews 2002; The Cochrane Library, Issue 3, 2002.

Bell-Syer SEM, Hart R, Crawford F, Torgerson DJ, Tyrrell W, Russell I. Oral treatments for fungal infections of the skin of the foot. Cochrane Database of Systematic Reviews 2002; The Cochrane Library, Issue 3, 2002.

Blake D, Proctor M, Johnson N, Olive D. Cleavage stage versus blastocyst stage embryo transfer in assisted conception. Cochrane Database of Systematic Reviews 2002; The Cochrane Library, Issue 3, 2002.

Bonfill X, Serra C, Sacristan M, Nogue M, Losa F, Montesinos J. Second-line chemotherapy for non-small cell lung cancer. Cochrane Database of Systematic Reviews 2002; The Cochrane Library, Issue 3, 2002.

Bowen A, Lincoln NB, Dewey M. Cognitive rehabilitation for spatial neglect following stroke. Cochrane Database of Systematic Reviews 2002; The Cochrane Library, Issue 3, 2002.

Buchbinder R, Green S, White M, Barnsley L, Smidt N, Assendelft WJJ. Shock wave therapy for lateral elbow pain. Cochrane Database of Systematic Reviews 2002; The Cochrane Library, Issue 3, 2002.

Cody J, Daly C, Campbell M, Donaldson C, Grant A, Khan I et al. Recombinant human erythropoietin for chronic renal failure anaemia in pre-dialysis patients. Cochrane Database of Systematic Reviews 2002; The Cochrane Library, Issue 3, 2002.

Cosmi B, Rubboli A, Castelvetri C, Milandri M. Ticlopidine versus oral anticoagulation for coronary stenting. Cochrane Database of Systematic Reviews 2002; The Cochrane Library, Issue 3, 2002.

Crawford F, Hart R, Bell-Syer S, Torgerson D, Young P, Russell I. Topical treatments for fungal infections of the skin and nails of the foot. Cochrane Database of Systematic Reviews 2002; The Cochrane Library, Issue 3, 2002.

Croft AMJ, Garner P. Mefloquine for preventing malaria in non-immune adult travellers. Cochrane Database of Systematic Reviews 2002; The Cochrane Library, Issue 3, 2002.

Dickersin K, Manheimer E. Surgery for nonarteritic anterior ischemic optic neuropathy. Cochrane Database of Systematic Reviews 2002; The Cochrane Library, Issue 3, 2002.

Dickson R, Awasthi S, Demellweek C, Williamson P. Anthelmintic drugs for treating worms in children: effects on growth and cognitive performance. Cochrane Database of Systematic Reviews 2002; The Cochrane Library, Issue 3, 2002.

Dinh-Zarr T, DiGuiseppi C, Heitman E, Roberts I. Interventions for preventing injuries in problem drinkers. Cochrane Database of Systematic Reviews 2002; The Cochrane Library, Issue 3, 2002.

Durkan A, Hodson EM, Willis NS, Craig JC. Non-corticosteroid treatment for nephrotic syndrome in children. Cochrane Database of Systematic Reviews 2002; The Cochrane Library, Issue 3, 2002.

Esposito M, Worthington HV, Coulthard P, Jokstad A. Interventions for replacing missing teeth: maintaining and re-establishing healthy tissues around dental implants. Cochrane Database of Systematic Reviews 2002; The Cochrane Library, Issue 3, 2002.

Evans JR, Henshaw K. Antioxidant vitamin and mineral supplementation for preventing age-related macular degeneration. Cochrane Database of Systematic Reviews 2002; The Cochrane Library, Issue 3, 2002.

Evans JR. Antioxidant vitamin and mineral supplements for age-related macular degeneration. Cochrane Database of Systematic Reviews 2002; The Cochrane Library, Issue 3, 2002.

Fenton M, Murphy B, Wood J, Bagnall AM, Chue P, Leitner M. Loxapine for schizophrenia. Cochrane Database of Systematic Reviews 2002; The Cochrane Library, Issue 3, 2002.

Fenton M, Morris S, DeSilva P, Bagnall AM, Cooper SJ, Gammelin G et al. Zotepine for schizophrenia. Cochrane Database of Systematic Reviews 2002; The Cochrane Library, Issue 3, 2002.

Furlan AD, Brosseau L, Irvin E. Massage for low back pain. Cochrane Database of Systematic Reviews 2002; The Cochrane Library, Issue 3, 2002.

Galandi D, Schwarzer G, Bassler D, Allgaier HP. Ursodeoxycholic acid and/or antibiotics for prevention of biliary stent occlusion. Cochrane Database of Systematic Reviews 2002; The Cochrane Library, Issue 3, 2002.

Galandi D, Antes G. Radiofrequency thermal ablation versus other interventions for hepatocellular carcinoma. Cochrane Database of Systematic Reviews 2002; The Cochrane Library, Issue 3, 2002.

Geddes JR, Freemantle N, Mason J, Eccles MP, Boynton J. Selective serotonin reuptake inhibitors (SSRIs) for depression. Cochrane Database of Systematic Reviews 2002; The Cochrane Library, Issue 3, 2002.

Gibbs S, Harvey I, Sterling JC, Stark R. Local treatments for cutaneous warts. Cochrane Database of Systematic Reviews 2002; The Cochrane Library, Issue 3, 2002.

Gibson JNA, Handoll HHG, Madhok R. Interventions for treating proximal humeral fractures in adults. Cochrane Database of Systematic Reviews 2002; The Cochrane Library, Issue 3, 2002.

Gilbody SM, Bagnall AM, Duggan L, Tuunainen A. Risperidone versus other atypical antipsychotic medication for schizophrenia. Cochrane Database of Systematic Reviews 2002; The Cochrane Library, Issue 3, 2002.

Gill D, Hatcher S. Antidepressants for depression in medical illness. Cochrane Database of Systematic Reviews 2002; The Cochrane Library, Issue 3, 2002.

Gillespie LD, Gillespie WJ, Robertson MC, Lamb SE, Cumming RG, Rowe BH. Interventions for preventing falls in elderly people. Cochrane Database of Systematic Reviews 2002; The Cochrane Library, Issue 3, 2002.

Green S, Buchbinder R, Barnsley L, Hall S, White M, Smidt N et al. Non-steroidal anti-inflammatory drugs for tennis elbow. Cochrane Database of Systematic Reviews 2002; The Cochrane Library, Issue 3, 2002.

Green S, Buchbinder R, Glazier R, Forbes A. Interventions for shoulder pain. Cochrane Database of Systematic Reviews 2002; The Cochrane Library, Issue 3, 2002.

Green S, Buchbinder R, Hall S, Barnsley L, Forbes A, Smidt N et al. Shock wave therapy for lateral elbow pain in adults. Cochrane Database of Systematic Reviews 2002; The Cochrane Library, Issue 3, 2002.

Green S, Buchbinder R, Barnsley L, Hall S, White M, Smidt N et al. Acupuncture for lateral elbow pain. Cochrane Database of Systematic Reviews 2002; The Cochrane Library, Issue 3, 2002.

Green S, Buchbinder R, Barnsley L, Hall S, White M, Smidt N et al. Non-steroidal anti-inflammatory drugs (NSAIDs) for treating lateral elbow pain in adults. Cochrane Database of Systematic Reviews 2002; The Cochrane Library, Issue 3, 2002.

Hagen KB, Hilde G, Jamtvedt G, Winnem M. Bed rest for acute low back pain and sciatica. Cochrane Database of Systematic Reviews 2002; The Cochrane Library, Issue 3, 2002.

Handoll HHG, Madhok R, Dodds C. Anaesthesia for treating distal radial fracture in adults. Cochrane Database of Systematic Reviews 2002; The Cochrane Library, Issue 3, 2002.

Handoll HHG, Rowe BH, Quinn KM, de Bie R. Interventions for preventing ankle ligament injuries. Cochrane Database of Systematic Reviews 2002; The Cochrane Library, Issue 3, 2002.

Handoll HHG, Madhok R. Surgical interventions for treating distal radial fractures in adults. Cochrane Database of Systematic Reviews 2002; The Cochrane Library, Issue 3, 2002.

Handoll HHG, Madhok R, Howe TE. Rehabilitation for distal radial fractures in adults. Cochrane Database of Systematic Reviews 2002; The Cochrane Library, Issue 3, 2002.

Handoll HHG, Madhok R. Conservative interventions for treating distal radial fractures in adults. Cochrane Database of Systematic Reviews 2002; The Cochrane Library, Issue 3, 2002.

Hilde G, Hagen KB, Jamtvedt G, Winnem M. Advice to stay active as a single treatment for low back pain and sciatica. Cochrane Database of Systematic Reviews 2002; The Cochrane Library, Issue 3, 2002.

Hiller JE, Griffith E, Jenner F. Education for contraceptive use by women after childbirth. Cochrane Database of Systematic Reviews 2002; The Cochrane Library, Issue 3, 2002.

Hilten J.J. v, Ramaker C, Beek WJTvd, Finken MJJ. Bromocriptine for levodopa-induced motor complications in Parkinson's disease. Cochrane Database of Systematic Reviews 2002; The Cochrane Library, Issue 3, 2002.

Hilton M, Pinder D. The Epley (canalith repositioning) manoeuvre for benign paroxysmal positional vertigo. Cochrane Database of Systematic Reviews 2002; The Cochrane Library, Issue 3, 2002.

Hodson EM, Knight JF, Willis NS, Craig JC. Corticosteroid therapy for nephrotic syndrome in children. Cochrane Database of Systematic Reviews 2002; The Cochrane Library, Issue 3, 2002.

Karjalainen K, Malmivaara A, van Tulder M, Roine R, Jauhiainen M, Hurri H et al. Multidisciplinary biopsychosocial rehabilitation for subacute low back pain among working age adults. Cochrane Database of Systematic Reviews 2002; The Cochrane Library, Issue 3, 2002.

Karjalainen K, Hurri H, Jauhiainen M, Koes BW, Malmivaara A, Roine R et al. Multidisciplinary rehabilitation for fibromyalgia and musculoskeletal pain in working age adults. Cochrane Database of Systematic Reviews 2002; The Cochrane Library, Issue 3, 2002.

Karjalainen K, Malmivaara A, van Tulder M, Roine R, Jauhiainen M, Hurri H et al. Multidisciplinary biopsychosocial rehabilitation for neck and shoulder pain among working age adults. Cochrane Database of Systematic Reviews 2002; The Cochrane Library, Issue 3, 2002.

Karjalainen K, Malmivaara A, van Tulder M., Roine R, Jauhiainen M, Hurri H et al. Biopsychosocial rehabilitation for upper limb repetitive strain injuries in working age adults. Cochrane Database of Systematic Reviews 2002; The Cochrane Library, Issue 3, 2002.

Lee A, Cooper MC, Craig JC, Knight JF, Keneally JP. Effects of nonsteroidal anti-inflammatory drugs on post-operative renal function in normal adults. Cochrane Database of Systematic Reviews 2002; The Cochrane Library, Issue 3, 2002.

Lewis R, Bagnall AM, Leitner M. Sertindole for schizophrenia. Cochrane Database of Systematic Reviews 2002; The Cochrane Library, Issue 3, 2002.

Leyland M, Zinicola E. Multifocal versus monofocal intraocular lenses after cataract extraction. Cochrane Database of Systematic Reviews 2002; The Cochrane Library, Issue 3, 2002.

Long V, Chen S. Surgical interventions for bilateral congenital cataract. Cochrane Database of Systematic Reviews 2002; The Cochrane Library, Issue 3, 2002.

Lutters M, Vogt N. Antibiotic duration for treating uncomplicated, symptomatic lower urinary tract infections in elderly women. Cochrane Database of Systematic Reviews 2002; The Cochrane Library, Issue 3, 2002.

Mabey D, Fraser-Hurt N. Antibiotics for trachoma. Cochrane Database of Systematic Reviews 2002; The Cochrane Library, Issue 3, 2002.

Macbeth F, Toy E, Coles B, Melville A, Eastwood A. Palliative radiotherapy regimens for non-small cell lung cancer. Cochrane Database of Systematic Reviews 2002; The Cochrane Library, Issue 3, 2002.

MacLeod A, Daly C, Khan I, Vale L, Campbell M, Wallace S et al. Cellulose, modified cellulose and synthetic membranes in the haemodialysis of patients with end-stage renal disease. Cochrane Database of Systematic Reviews 2002; The Cochrane Library, Issue 3, 2002.

Malthaner R, Fenlon D. Preoperative chemotherapy for resectable thoracic esophageal cancer. Cochrane Database of Systematic Reviews 2002; The Cochrane Library, Issue 3, 2002.

Manyemba J, Mayosi BM. Penicillin for secondary prevention of rheumatic fever. Cochrane Database of Systematic Reviews 2002; The Cochrane Library, Issue 3, 2002.

Martin-Hirsch P, Jarvis G, Kitchener H, Lilford R. Progestagens for endometrial cancer. Cochrane Database of Systematic Reviews 2002; The Cochrane Library, Issue 3, 2002.

McLauchlan GJ, Handoll HHG. Interventions for treating acute and chronic Achilles tendinitis. Cochrane Database of Systematic Reviews 2002; The Cochrane Library, Issue 3, 2002.

McNaughton Collins M, MacDonald R, Wilt T. Interventions for chronic abacterial prostatitis. Cochrane Database of Systematic Reviews 2002; The Cochrane Library, Issue 3, 2002.

Meremikwu M, Marson AG. Routine anticonvulsants for treating cerebral malaria. Cochrane Database of Systematic Reviews 2002; The Cochrane Library, Issue 3, 2002.

Moayyedi P, Soo S, Delaney B, Harris A, Innes M, Oakes R et al. Eradication of Helicobacter pylori for non-ulcer dyspepsia. Cochrane Database of Systematic Reviews 2002; The Cochrane Library, Issue 3, 2002.

Moayyedi P, Soo S, Deeks J, Delaney B, Innes M, Forman D. Pharmacological interventions for non-ulcer dyspepsia. Cochrane Database of Systematic Reviews 2002; The Cochrane Library, Issue 3, 2002.

Moyer VA, Craig JC. Short versus standard duration therapy for acute urinary tract infection in children. Cochrane Database of Systematic Reviews 2002; The Cochrane Library, Issue 3, 2002.

Mulrow C, Lau J, Cornell J, Brand M. Pharmacotherapy for hypertension in the elderly. Cochrane Database of Systematic Reviews 2002; The Cochrane Library, Issue 3, 2002.

Mulrow CD, Chiquette E, Angel L, Cornell J, Summerbell C, Anagnostelis B et al. Dieting to reduce body weight for controlling hypertension in adults. Cochrane Database of Systematic Reviews 2002; The Cochrane Library, Issue 3, 2002.

Oakley-Browne MA, Adams P, Mobberley PM. Interventions for pathological gambling. Cochrane Database of Systematic Reviews 2002; The Cochrane Library, Issue 3, 2002.

Parker MJ, Gillespie LD, Gillespie WJ. Hip protectors for preventing hip fractures in the elderly. Cochrane Database of Systematic Reviews 2002; The Cochrane Library, Issue 3, 2002.

Parkes J, Shepperd S. Discharge planning from hospital to home. Cochrane Database of Systematic Reviews 2002; The Cochrane Library, Issue 3, 2002.

Pearce PK, Handoll HHG, Der Tavitian A. Interventions for isolated diaphyseal fractures of the ulna in adults. Cochrane Database of Systematic Reviews 2002; The Cochrane Library, Issue 3, 2002.

Pratt BM, Woolfenden SR. Interventions for preventing eating disorders in children and adolescents. Cochrane Database of Systematic Reviews 2002; The Cochrane Library, Issue 3, 2002.

Richter B, Neises G. 'Human' insulin versus animal insulin in people with diabetes mellitus. Cochrane Database of Systematic Reviews 2002; The Cochrane Library, Issue 3, 2002.

Sheikh A, Hurwitz B, Cave J. Antibiotics versus placebo for acute bacterial conjunctivitis. Cochrane Database of Systematic Reviews 2002; The Cochrane Library, Issue 3, 2002.

Shelley MD, Barber J, Wilt T, Mason MD. Surgery versus radiotherapy for muscle invasive bladder cancer. Cochrane Database of Systematic Reviews 2002; The Cochrane Library, Issue 3, 2002.

Shelley MD, Court JB, Kynaston H, Wilt TJ, Fish RG, Mason M. Intravesical Bacillus Calmette-Guerin in Ta and T1 Bladder Cancer. Cochrane Database of Systematic Reviews 2002; The Cochrane Library, Issue 3, 2002.

Shepherd J, Weston R, Peersman G, Napuli IZ. Interventions for encouraging sexual lifestyles and behaviours intended to prevent cervical cancer. Cochrane Database of Systematic Reviews 2002; The Cochrane Library, Issue 3, 2002.

Shepperd S, Iliffe S. Hospital at home versus in-patient hospital care. Cochrane Database of Systematic Reviews 2002; The Cochrane Library, Issue 3, 2002.

Shey Wiysonge CU, Brocklehurst P, Sterne JAC. Vaginal disinfection during labour for reducing the risk of mother-to-child transmission of HIV infection. Cochrane Database of Systematic Reviews 2002; The Cochrane Library, Issue 3, 2002.

Smeeth L, Iliffe S. Community screening for visual impairment in the elderly. Cochrane Database of Systematic Reviews 2002; The Cochrane Library, Issue 3, 2002.

Snellingen T, Evans JR, Ravilla T, Foster A. Surgical interventions for age-related cataract. Cochrane Database of Systematic Reviews 2002; The Cochrane Library, Issue 3, 2002.

Snowden HM, Renfrew MJ, Woolrdige MW. Treatments for breast engorgement during lactation. Cochrane Database of Systematic Reviews 2002; The Cochrane Library, Issue 3, 2002.

Srisurapanont M, Jarusuraisin N. Opioid antagonists for alcohol dependence. Cochrane Database of Systematic Reviews 2002; The Cochrane Library, Issue 3, 2002.

Struijs PAA, Smidt N, Arola H, Dijk van CN, Buchbinder R, Assendelft WJJ. Orthotic devices for the treatment of tennis elbow. Cochrane Database of Systematic Reviews 2002; The Cochrane Library, Issue 3, 2002.

Towheed T, Shea B, Wells G, Hochberg M. Analgesia and non-aspirin, non-steroidal anti-inflammatory drugs for osteoarthritis of the hip. Cochrane Database of Systematic Reviews 2002; The Cochrane Library, Issue 3, 2002.

Van Perperstraten AM, Proctor ML, illipson G, ohnson NP. Techniques for surgical retrieval of sperm prior to ICSI for azoospermia. Cochrane Database of Systematic Reviews 2002; The Cochrane Library, Issue 3, 2002.

van Tulder M, Ostelo RWJG, Vlaeyen JWS, Linton SJ, Morley SJ, Assendelft WJJ. Behavioural treatment for chronic low back pain. Cochrane Database of Systematic Reviews 2002; The Cochrane Library, Issue 3, 2002.

Vimalachandra D, Craig JC, Cowell C, Knight JF. Growth hormone for children with chronic renal failure. Cochrane Database of Systematic Reviews 2002; The Cochrane Library, Issue 3, 2002.

Wilhelmus KR. Interventions for herpes simplex virus epithelial keratitis. Cochrane Database of Systematic Reviews 2002; The Cochrane Library, Issue 3, 2002.

Wilkins M, Indar A, Wormald R. Intra-operative Mitomycin C for glaucoma surgery. Cochrane Database of Systematic Reviews 2002; The Cochrane Library, Issue 3, 2002.

Williams GJ, Lee A, Craig JC. Long-term antibiotics for preventing recurrent urinary tract infection in children. Cochrane Database of Systematic Reviews 2002; The Cochrane Library, Issue 3, 2002.

Williams JW, Aguilar C, Makela M, Cornell J, Hollman DR, Chiquette E et al. Antibiotics for acute maxillary sinusitis. Cochrane Database of Systematic Reviews 2002; The Cochrane Library, Issue 3, 2002.

Wilson K, Mottram P, Sivanranthan A, Nightingale A. Antidepressants versus placebo for the depressed elderly. Cochrane Database of Systematic Reviews 2002; The Cochrane Library, Issue 3, 2002.

Wilt T, Ishani A, MacDonald R, Stark G, Mulrow C, Lau J. Beta-sitosterols for benign prostatic hyperplasia. Cochrane Database of Systematic Reviews 2002; The Cochrane Library, Issue 3, 2002.

Wilt T, MacDonald R, Ishani A, Rutks I, Stark G. Cernilton for benign prostatic hyperplasia. Cochrane Database of Systematic Reviews 2002; The Cochrane Library, Issue 3, 2002.

Wilt T, Ishani A, MacDonald R. Serenoa repens for benign prostatic hyperplasia. Cochrane Database of Systematic Reviews 2002; The Cochrane Library, Issue 3, 2002.

Wilt T, Ishani A, MacDonald R, Rutks I, Stark G. Pygeum africanum for benign prostatic hyperplasia. Cochrane Database of Systematic Reviews 2002; The Cochrane Library, Issue 3, 2002.

Wilt TJ, MacDonald R, Rutks I, Howe RW, Chapple C. Tamsulosin for benign prostatic hyperplasia. Cochrane Database of Systematic Reviews 2002; The Cochrane Library, Issue 3, 2002.

Wong R, Malthaner R. Combined chemotherapy and radiotherapy (without surgery) compared with radiotherapy alone in localized carcinoma of the esophagus. Cochrane Database of Systematic Reviews 2002; The Cochrane Library, Issue 3, 2002.

Wormald R, Wilkins MR, Bunce C. Post-operative 5-Fluorouracil for glaucoma surgery. Cochrane Database of Systematic Reviews 2002; The Cochrane Library, Issue 3, 2002.

Yeung EW, Yeung SS. Interventions for preventing lower limb soft-tissue injuries in runners. Cochrane Database of Systematic Reviews 2002; The Cochrane Library, Issue 3, 2002.
